# Topical Tacrolimus in the Management of High-Risk Keratoplasty: A Systematic Review and Meta-Analysis

**DOI:** 10.7759/cureus.82591

**Published:** 2025-04-19

**Authors:** Abdulmajeed Al Khathami, Ruba A Alamri, Reem A Garah, Rawan S Alsamli, Nourah B Alsheikh, Omar B Alsheikh Alshahrani, Yazeed S AlHoshan, Sarah M Alaklabi, Shaima A Benyh, Abdulrahman Alamri

**Affiliations:** 1 Department of Ophthalmology, King Fahad Hospital, Al Baha Health Cluster, Al Baha, SAU; 2 Medicine and Surgery, Taibah University, Medina, SAU; 3 College of Medicine, Taibah University, Al Madinah Al Monawarah, SAU; 4 College of Medicine and Surgery, Umm Al-Qura University, Makkah, SAU; 5 College of Medicine, University of Bisha, Bisha, SAU; 6 College of Medicine, King Saud Bin Abdulaziz University for Health Sciences College of Medicine, Riyadh, SAU; 7 Ophthalmology, College of Medicine, King Khalid University, Abha, SAU

**Keywords:** corneal transplantation, high-risk keratoplasty, systematic review and meta-analysis, tacrolimus, therapeutic penetrating keratoplasty

## Abstract

Corneal transplantation is a frequently performed procedure for human eye transplants. In low-risk conditions such as keratoconus and Fuch's dystrophy, corneal transplantation has a high success rate due to the immune advantages in support of the cornea. Major risk factors for corneal graft rejection include: atopy, chemical burn, prolonged herpes infection, infectious leukoma, trauma, prior transplantation, active inflammation, and corneal neovascularization. Treatments against lymphangiogenic bacteria and anti-angiogenic bacteria are two strategies to reduce corneal graft rejection. The primary objective of this systematic review and meta-analysis was to assess topical tacrolimus's benefits for high-risk keratoplasty management.

In accordance with Preferred Reporting Items for Systematic Reviews and Meta-Analyses (PRISMA) guidelines, data was gathered by combining pertinent medical subject words in the literature of the complementing articles from the PubMed, Cochrane Library, Embase, Web of Science, Medline, and Google Scholar. The search was conducted using terms such as "tacrolimus," "keratoplasty," and other related synonymous terms. Prior to conducting a meta-analysis, Review Manager version 5.3.2 (www.revman.cochrane.org) was utilized to assess the risk of bias in the extracted data, and the literature was chosen according to pre-established inclusion and exclusion criteria. The meta-analysis employed both Forest plots and funnel plots to examine heterogeneity and assess the likelihood of publication bias. Eight studies were included in the analysis. The results indicated a moderate level of heterogeneity among the included studies, with Tau2 = 0.19 and I2 = 52%. Moreover, the overall findings suggested that topical tacrolimus treatment emerged as the most effective medication for high-risk keratoplasty, as evidenced by the diamond marker positioned slightly to the left side of the 95% confidence interval (0.67; 0.42-1.06), rather than the right side. Subgroup analysis further reinforced this notion, showing that topical tacrolimus was particularly efficacious in treating high-risk keratoplasty, with a 95% confidence interval of (0.16; -0.68-1.00). However, the difference observed was minimal, possibly due to certain studies comparing topical tacrolimus with mixed treatment doses, some of which included a combination of tacrolimus and other agents. Additionally, topical tacrolimus treatment was identified as the safest option for high-risk keratoplasty, with a confidence interval of (-0.35; -2.23-0.88). Nonetheless, the overall effect suggested that the medication did not significantly differ from other treatments. This study confirms that topical tacrolimus is both effective and the safest treatment for managing high-risk penetrating keratoplasty (PKP). However, future studies should focus on determining the appropriate dosage levels for the management of high-risk PKP.

## Introduction and background

The most common type of human eye transplantation is corneal transplantation. The cornea's immune privilege, a unique immunological status that helps it evade immune system attacks, grafts typically succeed in low-risk scenarios such as keratoconus or Fuchs' endothelial dystrophy [1]. However, this immune privilege is not absolute. Transplant rejection continues to pose a major challenge, especially in high-risk recipients. However, immune privilege alone cannot prevent transplant rejection, and the rate of high-risk graft failure is increasing. Some of the major risk factors for corneal graft rejection include: atopy, chemical burn, prolonged herpes infection, infectious leukoma, trauma, prior transplantation, active inflammation, and corneal neovascularization. These conditions significantly elevate the risk of immune rejection and subsequent graft failure. In some cases, rejection rates may exceed 60%, despite modern surgical and medical advances [2].

Treatments against lymphangiogenic bacteria and anti-angiogenic bacteria are two strategies to reduce corneal graft rejection [3]. In addition to standard therapy using topical steroids to reduce transplant rejection and increase transplant survival rates, topical tacrolimus and cyclosporine serve as helpful supplements. For graft survival, tacrolimus, mycophenolate, and systemic cyclosporine are all advantageous [4]. In order to help patients with corneal blindness regain their visual acuity, corneal transplantation is now considered an effective treatment option. Normal corneal tissues lack lymphatic and blood vessels, which significantly reduces the incidence of immune rejection when compared to organ transplantation [5]. However, the incidence of rejection often exceeds 60%, and the graft survival rate is notably lower for patients undergoing high-risk corneal transplantation, such as those with chemical burns, multiple or bilateral corneal transplantation, and children [6]. Consequently, there is an urgent need for a novel anti-immune rejection treatment strategy.

Glucocorticoids are known to be an essential medication in the anti-rejection therapy used after corneal transplantation, acting to both prevent and treat immune rejection events [7]. However, due to side effects like cataract formation and hormonal glaucoma, as well as the acceleration or worsening of infections, glucocorticoids are frequently used in combination with other pharmaceutical agents due to their prolonged administration [8]. Combining cyclosporine eye drops with glucocorticoids has proven to be beneficial, but most patients experience noticeable ocular irritation symptoms after treatment, which results in less than ideal adherence [9]. Research has demonstrated that short-term systemic cyclosporine treatment significantly reduces the incidence of postoperative rejection in high-risk corneal transplant recipients. However, hepatotoxicity, nephrotoxicity, hypertension, and other side effects are frequently experienced after use, making it unsuitable for prolonged use [10]. It has recently been demonstrated that the immunosuppressive medication tacrolimus has a potent immunosuppressive effect. Tacrolimus eye drops administered locally have been shown in numerous studies to have positive effects on high-risk corneal transplant recipients. However, the rejection rate stands at about 16% [11]. Research has also indicated that tropical tacrolimus therapy, when administered to high-risk corneal transplant recipients who have not responded to cyclosporine antirejection treatment, is a safe and effective method to prolong graft survival and decrease corneal graft rejection [12]. Therefore, the goal of this meta-analysis and systematic review is to demonstrate the benefits of topical tacrolimus in the treatment of high-risk keratoplasty.

## Review

Materials and methods

In accordance with Preferred Reporting Items for Systematic Reviews and Meta-Analyses (PRISMA) guidelines, data was gathered by combining pertinent medical subject words in the literature of the complementing articles from the PubMed, Cochrane Library, Embase, Web of Science, Medline, and Google Scholar. The search was conducted using terms such as "tacrolimus," "keratoplasty," and other related synonymous terms. Boolean logic operations were used to combine the results. Due to limited randomized controlled trials (RCTs) on the subject matter, the search focused on the studies conducted from March 2013 to March 2024.

Criteria for literature selection 

Inclusion Criteria 

The following criteria were applied when choosing research articles for this meta-analysis: articles published in English-language journals with topical tacrolimus as a treatment of penetrating keratoplasty; randomized controlled trials (RCTs); articles that have reported primary or secondary outcome indicators.

Exclusion Criteria 

Articles written in languages other than English, trials involving subjects other than humans, reports lacking primary or secondary outcome indicators, studies with unclear or insufficient data, observational studies, non-randomized controlled trials, editorial publications, and articles with only published abstracts were all excluded. 

Data Extraction and Management 

Two independent experts reviewed the original studies. Research with conflicting reviewers' conclusions was either discussed to reach a consensus or forwarded to a third reviewer for confirmation. The initial author, the year of release, the nation of origin, the sample size, the study design, the interventions, the inclusion criteria, and the research findings were all included in each study.

Statistical analysis**

The data analysis program utilized was Review Manager version 5.3.2 (www.revman.cochrane.org). The quantitative analyses included the sample size, mean, and standard deviation (SD) for every outcome. To determine the degree of similarity, results from evaluation scales that were similar were reanalyzed using the weighted mean difference (WMD) and 95% confidence interval (CI). In instances where a noteworthy distinction existed between the evaluation scales, 95% confidence intervals and standard mean differences (SMD) were employed. To evaluate the heterogeneity among the trials that were included, I2 test statistics were employed. When there was clear heterogeneity and an I2 >50%, a fixed effect model was used; otherwise, a random effect model was used. There was a p<0.05 significance level applied. Additionally, a funnel plot was used to investigate publication bias. 

Results

**Figure 1:**
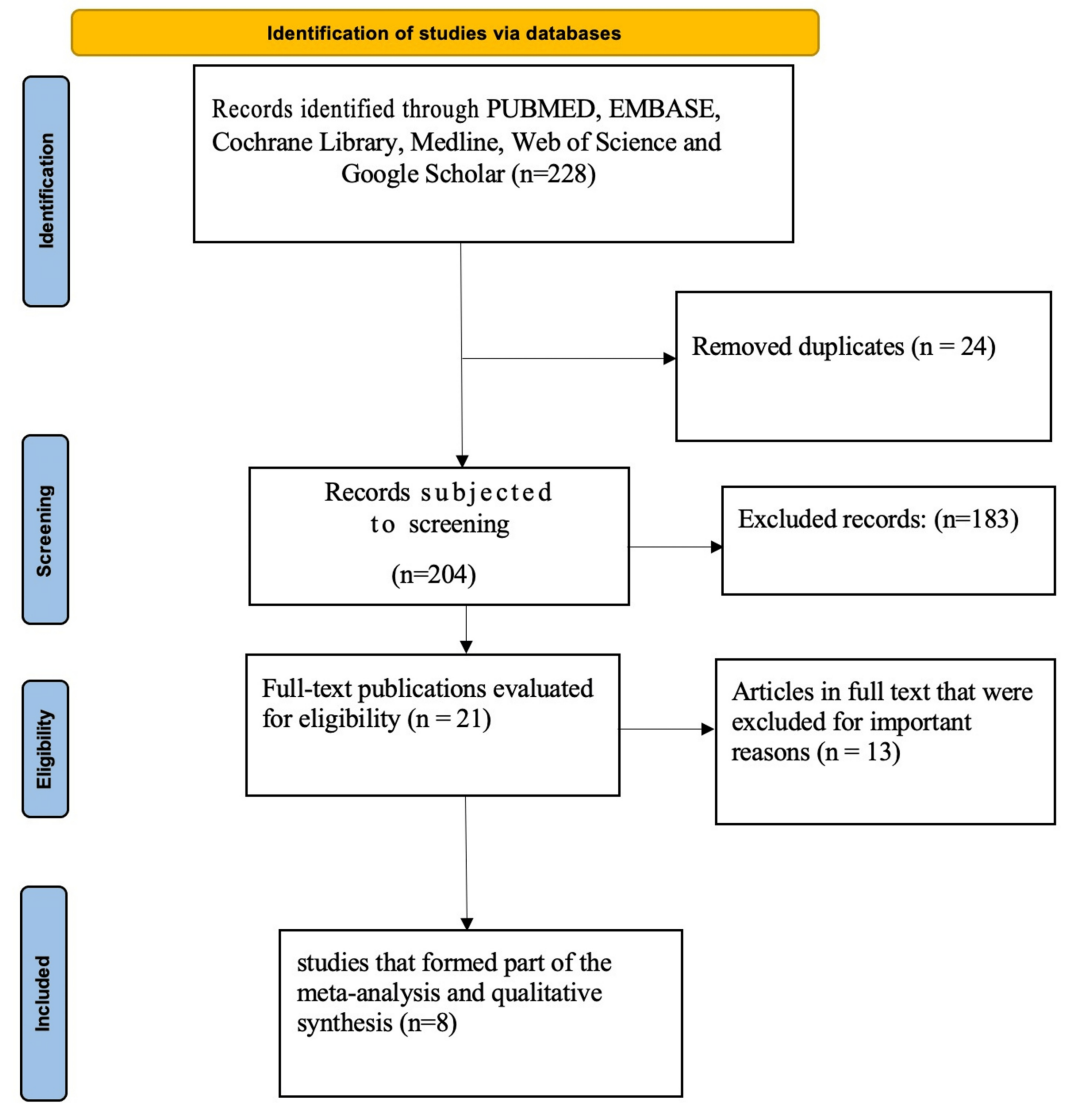
PRISMA Flow Diagram PRISMA: Preferred Reporting Items for Systematic Reviews and Meta-Analyses

The database was searched using the previously mentioned methodology, which yielded a total of 228 articles, of which 110 came from PubMed, 18 from Embase, 24 from the Cochrane Library, 26 from Medline, 27 from Web of Science, and 23 from Google Scholar. Following a rigorous screening process and eligibility checks in accordance with pre-established inclusion and exclusion criteria, eight articles were ultimately included in the qualitative synthesis and meta-analysis by PRISMA flow diagram as displayed in Figure 1.

Study Characteristics

The key features of the included studies are displayed in Table 1. This study examined topical tacrolimus treatment of high-risk keratoplasty in all eight articles included. All eight articles were RCTs published in English-language journals in different regions across the world. There were 459 patients in the total combined sample size. The characteristics of each study are shown across the rows, while general characteristics of the studies are shown down the columns.

**Table 1:** Characteristics of the included studies HSK-herpes simplex keratitis; RCT-randomized controlled trials, PKP-penetrating keratoplasty; GVHD-graft versus host disease; CsA-Central sleep Apnea; MMF-Mycophenolate mofetil.

Author	Region	Sample size	Study design	Intervention	Inclusion	Outcome/Results.
Akbari et al., 2019 [13]	Iran	50	Randomized clinical trial	Tacrolimus	English-language publications with topical tacrolimus as the treatment of penetrating keratoplasty; randomised controlled trials (RCTs); having report of primary or secondary outcome indicators	Topical tacrolimus (0.05%) is an appropriate adjunct therapy for patients with HSK because it improves visual acuity and decreases corneal inflammation, neovascularization, and scarring.
Zhai et al., 2020 [14]	North China	49	Randomized clinical trial	Tacrolimus	English-language publications with topical tacrolimus as the treatment of penetrating keratoplasty; randomised controlled trials (RCTs); having report of primary or secondary outcome indicators	It was discovered that high-risk PKP patients responded very well to topical tacrolimus 01% treatment, as it had fewer side effects than topical cyclosporine 1%, and it significantly reduced corneal graft rejection.
Chin et al., 2021 [15]	Korea	144	Randomized control trial	0.05 mg/kg tacrolimus	English-language publications with topical tacrolimus as the treatment of penetrating keratoplasty; randomised controlled trials (RCTs); having report of primary or secondary outcome indicators	Compared to tapered steroids alone, When taking tacrolimus (3–8 ng/ml) for maintenance, considerably fewer patients experienced relapses (5.7% versus 22.6%, respectively; P=0.01).
Faramarzi et al., 2021 [16]	Tehran, Iran	63	Randomized clinical trial	1 g tacrolimus	English-language publications with topical tacrolimus as the treatment of penetrating keratoplasty; randomised controlled trials (RCTs); having report of primary or secondary outcome indicators	On a 12-month follow-up after repeat keratoplasty, topical 0.03% tacrolimus was found to be as effective as systemic MMF when used in conjunction with systemic and topical corticosteroids to reduce endothelial graft rejection.
Abud et al., 2016 [17]	Massachusetts. England	40	Randomized, double-masked clinical trial	Tacrolimus	English-language publications with topical tacrolimus as the treatment of penetrating keratoplasty; randomised controlled trials (RCTs); having report of primary or secondary outcome indicators	Topical tacrolimus 0.05% is an effective, safe, and generally well-tolerated treatment for ocular GVHD, as opposed to the hypertensive side effects of topical corticosteroids.
Hashemian et al., 2018 [18]	Iran	31	Randomized control trial	Tacrolimus at 0.05%	English-language publications with topical tacrolimus as the treatment of penetrating keratoplasty; randomised controlled trials (RCTs); having report of primary or secondary outcome indicators	PKP graft endothelial rejection can be resolved more quickly and may be less likely to recur when topical tacrolimus 0.05% is used in conjunction with steroids. The success of rejection reversal may not be enhanced by it, though.
Yamazoe et al., 2014 [19]	Japan	10	Randomized controlled trial	Systemic tacrolimus	English-language publications with topical tacrolimus as the treatment of penetrating keratoplasty; randomized controlled trials (RCTs); having report of primary or secondary outcome indicators	When systemic CsA induces graft failure in patients with high-risk PKP, systemic tacrolimus treatment may be safe and effective in reducing graft rejection and prolonging graft survival.
Magalhaes et al., 2013 [20]	Brazil	72	Retrospective Randomized control trial (RCT)	Tacrolimus	English-language publications with topical tacrolimus as the treatment of penetrating keratoplasty; randomized controlled trials (RCTs); having report of primary or secondary outcome indicators	Topical 0.03% tacrolimus prevents irreversible rejection without increasing intraocular pressure in patients undergoing high-risk corneal transplantation.

Quality Assessment of the Studies

**Figure 2:**
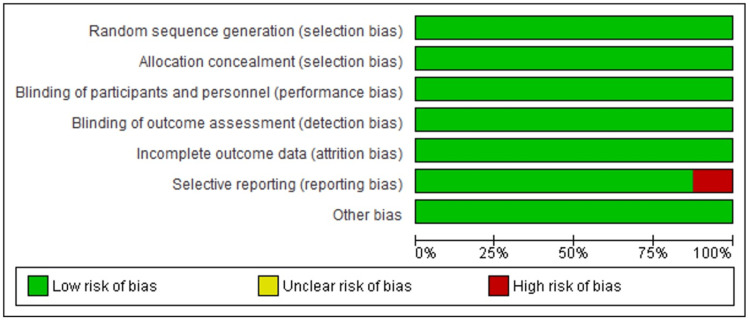
Risk of publication bias

The assessment in Figure 2 is based on the article's synopsis, whereby the graph's red area denotes a high risk of bias, the green area denotes a low risk, and the yellow area denotes an unclear risk of bias. In terms of selection, detection, attrition, and performance biases, most of the studies under review indicated a low risk of bias. Nonetheless, reporting bias was present in just 12.5% of the included studies. 

Meta-Analysis Outcome

**Figure 3:**
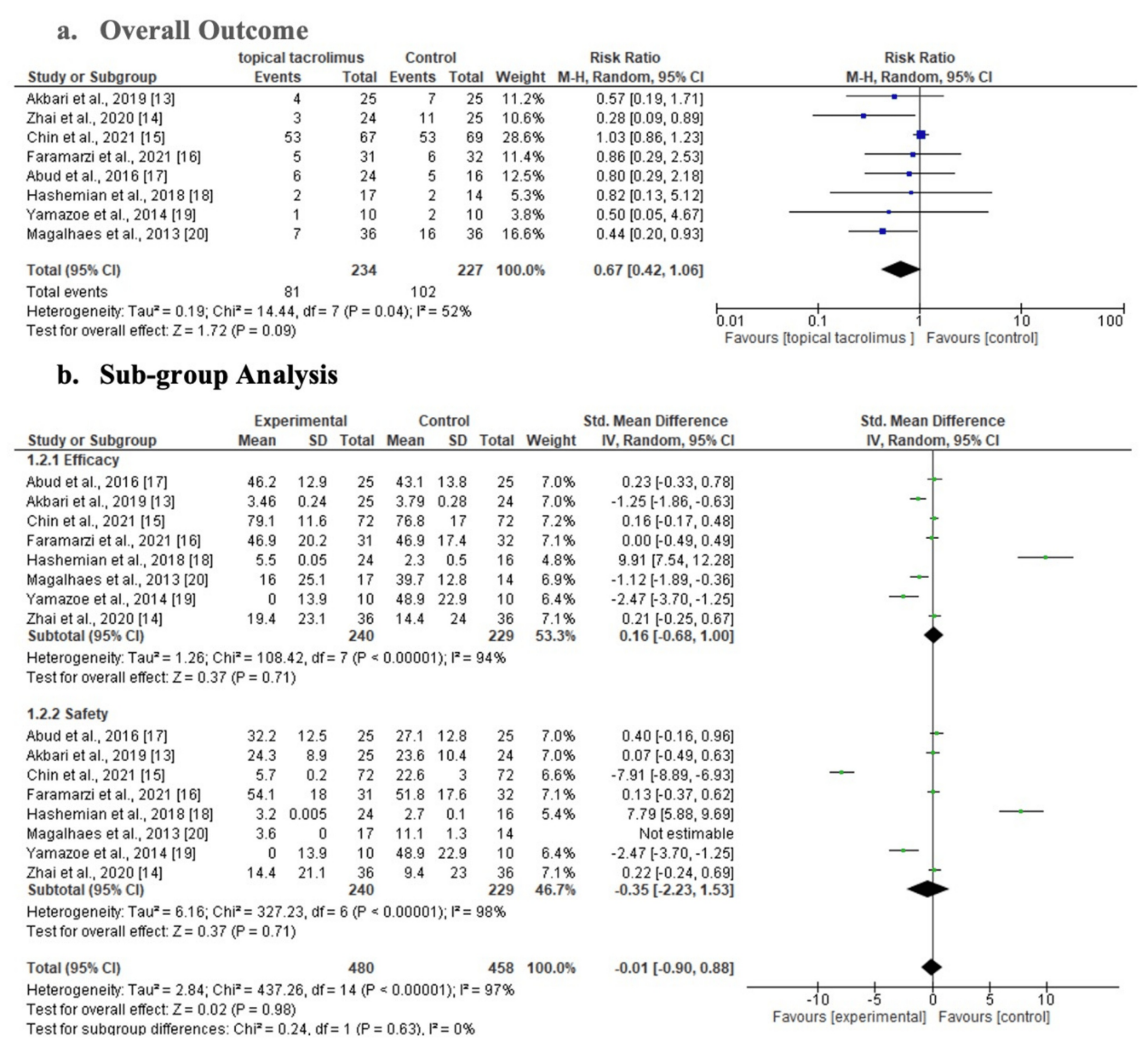
Forest plots of the outcome

The findings of the eight studies that were part of the investigation are displayed in Figure 3. The findings show that there was moderate heterogeneity among the included studies (Tau2= 0.19, I2= 52%). Similarly, the overall result indicates that topical tacrolimus treatment was the most effective medication for high-risk keratoplasty; this is demonstrated by the diamond marker, which at 95% CI (0.67; 0.42-1.06) lies slightly on the left side compared to the right. However, subgroup analysis demonstrates that topical tacrolimus is more effective in treating high-risk keratoplasty patients (95% CI: 0.16; -0.68-1.00). However, the difference was negligible because some studies compared the topical tacrolimus with a mixed treatment dose, in which a specific amount of topical tacrolimus was included in some doses. Not to be overlooked, topical tacrolimus treatment is found to be the safest option for high-risk keratoplasty (-0.35; -2.23-0.88). However, overall, the results indicate that the medication did not significantly differ from other treatments. This is likely due to the fact that some of the studies evaluated different dosage combinations.

Funnel Plots

**Figure 4:**
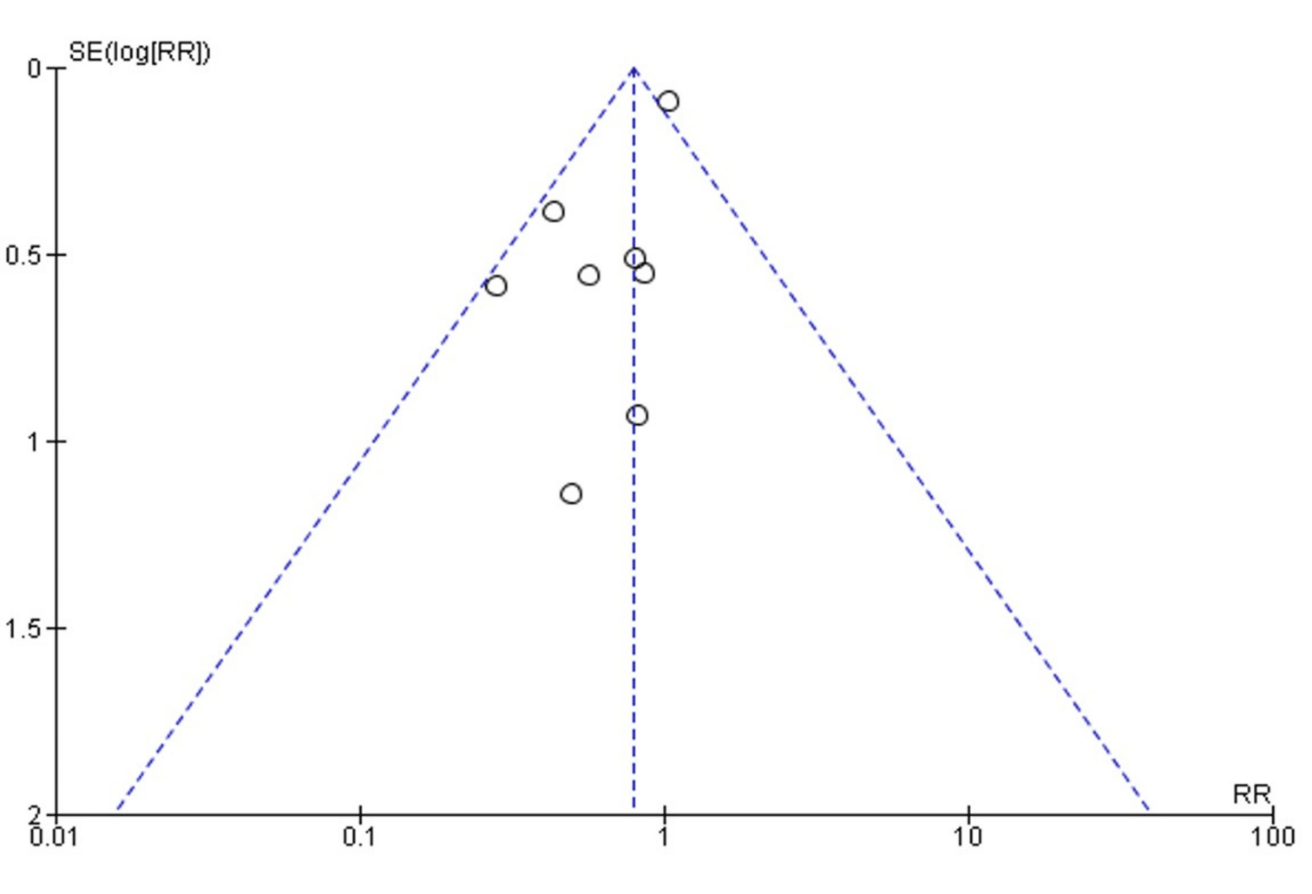
Funnel Plot for Publication Bias. X-Axis: Standard Error; Y-Axis: Effect Size; SE-standard error; RR-relative risk. Topical Tacrolimus group, Control group.

The funnel plot results in Figure 4 show that the studies were more aligned to the left as opposed to the right sides. This indicates a symmetric distribution of effect sizes as a function of study precision. The sampled studies' symmetrical distribution shows a possibility of publication bias to the left side. 

Discussion

The benefit of topical tacrolimus in the treatment of high-risk penetrating keratoplasty (PKP) is investigated in this study. The findings of the study reveal that the overall outcome shows that topical tacrolimus treatment proved to be the best medication for high risk keratoplasty as indicated by the diamond marker which lies partially on the left side as opposed to the right side based on the statistical test at 95% CI (0.67; 0.42-1.06). These findings concur with the results obtained in a study in Iran, which revealed that patients with herpes simplex keratitis (HSK), 0.05% topical tacrolimus can be added as an appropriate adjunct treatment because it improves visual acuity and decreases corneal inflammation, neovascularization, and scarring [13]. Furthermore, subgroup analysis indicates that topical tacrolimus is more efficient in treating high-risk keratoplasty, with a 95% CI (0.16; -0.68-1.00). However, the difference was minimal, as some studies compared topical tacrolimus with a mixed treatment dose, where certain doses included a combination of topical tacrolimus. These findings are consistent with the findings obtained in a study in northern China, which revealed that topical tacrolimus 01% is highly safe and effective treatment for high-risk penetrating keratoplasty (PKP) patients, as it had fewer side effects than topical cyclosporine 1%, and it significantly reduced corneal graft rejection [14]. In addition, a study in Korea noted that compared to tapered steroids alone, significantly fewer patients experienced relapses while receiving maintenance tacrolimus (3-8 ng/ml) (5.7% versus 22.6%, respectively; P=0.01) [15].

The study found that topical tacrolimus treatment is the safest option for high-risk keratoplasty (-0.35; -2.23-0.88). However, the overall effect demonstrated that the medication did not have a significant impact compared to other treatments, which can be attributed to the results of studies that utilized mixed doses with varying quantities of treatment being evaluated. In Tehran, Iran, a study found that during a 12-month follow-up after repeat keratoplasty, topical 0.03% tacrolimus was just as effective as systemic mycophenolate mofetil (MMF) when topical and systemic corticosteroids are used together to reduce endothelial graft rejection [16]. Additionally, a study by Abud et al. revealed that compared to hypertensive side effects of topical corticosteroids, topical tacrolimus 0.05% is a safe, generally well-tolerated, and effective treatment for ocular graft versus host disease (GVHD) [17].

A study conducted in Iran suggests that the resolution of PKP graft endothelial rejection may occur more rapidly and with reduced likelihood of recurrence when topical tacrolimus 0.05% is combined with steroids. However, the success of rejection reversal may not be enhanced by it, though [18]. Conversely, the study by Yamazoe et al. indicates that in patients with high-risk PKP, systemic tacrolimus treatment may be both safe and effective in reducing graft rejection and prolonging graft survival subsequent to systemic Central sleep Apnea (CsA)-induced graft failure [19]. To corroborate the results, Magalhaes et al. also discovered that topical 0.03% tacrolimus prevents irreversible rejection in patients receiving high-risk corneal transplantation without increasing intraocular pressure [20]. These results support the notion that topical tacrolimus is the safest and effective treatment for high-risk penetrating keratoplasty (PKP). The study did not, however, determine the dosage that can be applied to the management of penetrating keratoplasty (PKP) at high risk.

This study had some drawbacks: the sample size of the included studies was small, which might have subjected the study to be prone to biases; the analysis of the overall tacrolimus treatment revealed moderate level of heterogeneity in the combined literature data thus lowering its reliability in the broad context. Hence, more investigation will be required to confirm the study's findings.

## Conclusions

In conclusion, the study reveals that in comparison to other treatments, the topical tacrolimus is the most effective medication for high-risk keratoplasty. Subgroup analysis confirms the effectiveness of topical tacrolimus in treating high-risk keratoplasty. It was also discovered to be the most secure course of treatment for this illness. The different treatment dosages examined in the trials may have contributed to the overall effect's lack of a significant difference when compared to other treatments. Generally, the study points out that topical tacrolimus is a worthwhile medication for handling high-risk keratoplasty; however, more studies using standardized dosages are necessary to verify the most effective treatment dosage.

## References

[1] Birnbaum F, Mayweg S, Reis A (2009). Mycophenolate mofetil (MMF) following penetrating high-risk keratoplasty: long-term results of a prospective, randomised, multicentre study. Eye (Lond).

[2] Hill JC (1995). Systemic cyclosporine in high-risk keratoplasty: long-term results. Eye (Lond).

[3] Qi X, Wang L, Zhang X, Liu M, Gao H (2022). Topical administration of tacrolimus and corticosteroids in tapering doses is effective in preventing immune rejection in high-risk keratoplasty: a 5-year follow-up study. BMC Ophthalmol.

[4] Reinhard T, Mayweg S, Reis A, Sundmacher R (2005). Topical FK506 as immunoprophylaxis after allogeneic penetrating normal-risk keratoplasty: a randomized clinical pilot study. Transpl Int.

[5] Shimmura-Tomita M, Shimmura S, Satake Y, Shimazaki-Den S, Omoto M, Tsubota K, Shimazaki J (2013). Keratoplasty postoperative treatment update. Cornea.

[6] Jabbehdari S, Rafii AB, Yazdanpanah G, Hamrah P, Holland EJ, Djalilian AR (2017). Update on the management of high-risk penetrating keratoplasty. Curr Ophthalmol Rep.

[7] Mayer K, Reinhard T, Reis A, Voiculescu A, Sundmacher R (2003). Synergistic antiherpetic effect of acyclovir and mycophenolate mofetil following keratoplasty in patients with herpetic eye disease: first results of a randomised pilot study. Graefes Arch Clin Exp Ophthalmol.

[8] Dhaliwal JS, Mason BF, Kaufman SC (2008). Long-term use of topical tacrolimus (FK506) in high-risk penetrating keratoplasty. Cornea.

[9] Sloper CM, Powell RJ, Dua HS (2001). Tacrolimus (FK506) in the management of high-risk corneal and limbal grafts. Ophthalmology.

[10] Reinhard T, Mayweg S, Sokolovska Y (2005). Systemic mycophenolate mofetil avoids immune reactions in penetrating high-risk keratoplasty: preliminary results of an ongoing prospectively randomized multicentre study. Transpl Int.

[11] Bernardes L, Gil J, Costa E, Tavares C, Rosa A, Quadrado MJ, Murta J (2024). Topical tacrolimus in high-risk corneal transplants. Eur J Ophthalmol.

[12] Joseph A, Raj D, Shanmuganathan V, Powell RJ, Dua HS (2007). Tacrolimus immunosuppression in high-risk corneal grafts. Br J Ophthalmol.

[13] Akbari M, Soltani Moghadam R, Elmi R, Nosrati A, Taghiabadi E, Aghdami N (2019). Topical tacrolimus as an adjunct to conventional therapy for stromal herpetic keratitis: a randomized clinical trial. J Ophthalmic Vis Res.

[14] Zhai LY, Zhang XR, Liu H, Ma Y, Xu HC (2020). Observation of topical tacrolimus on high-risk penetrating keratoplasty patients: a randomized clinical trial study. Eye (Lond).

[15] Chin HJ, Chae DW, Kim YC (2021). Comparison of the efficacy and safety of tacrolimus and low-dose corticosteroid with high-dose corticosteroid for minimal change nephrotic syndrome in adults. J Am Soc Nephrol.

[16] Faramarzi A, Abbasi H, Feizi S (2021). Topical 0.03% tacrolimus versus systemic mycophenolate mofetil as adjuncts to systemic corticosteroids for preventing graft rejection after repeat keratoplasty: one-year results of a randomized clinical trial. Eye (Lond).

[17] Abud TB, Amparo F, Saboo US (2016). A clinical trial comparing the safety and efficacy of topical tacrolimus versus methylprednisolone in ocular graft-versus-host disease. Ophthalmology.

[18] Hashemian MN, Latifi G, Ghaffari R (2018). Topical tacrolimus as adjuvant therapy to corticosteroids in acute endothelial graft rejection after penetrating keratoplasty: a randomized controlled trial. Cornea.

[19] Yamazoe K, Yamazoe K, Yamaguchi T, Omoto M, Shimazaki J (2014). Efficacy and safety of systemic tacrolimus in high-risk penetrating keratoplasty after graft failure with systemic cyclosporine. Cornea.

[20] Magalhaes OA, Marinho DR, Kwitko S (2013). Topical 0.03% tacrolimus preventing rejection in high-risk corneal transplantation: a cohort study. Br J Ophthalmol.

